# Protopanaxadiol stimulates glucose consumption by modulating the AMP-activated protein kinase pathway in myotubes, hepatoma cells, and adipocytes

**DOI:** 10.1371/journal.pone.0328486

**Published:** 2025-07-29

**Authors:** Dahae Lee, Sang Hee Shim, Ki Sung Kang

**Affiliations:** 1 College of Korean Medicine, Gachon University, Seongnam, Republic of Korea; 2 Natural Products Research Institute, College of Pharmacy, Seoul National University, Seoul, South Korea; University of Bergen: Universitetet i Bergen, NORWAY

## Abstract

Ginsenosides, the main active constituents of *Panax ginseng*, possess potent anti-diabetic and anti-obesity properties. In this study, we investigated the molecular and cellular mechanisms underlying the effects of protopanaxadiol (PPD), Rg3, Rb2, Re, Rc, Rh2, Rb1, Rg1, and compound K on palmitic acid (PA)-induced lipid accumulation in HepG2 hepatoma cells and glucose consumption (GC) in C2C12 myotubes and 3T3-L1 adipocytes. PA-induced lipid accumulation was determined using lipid (Oil Red O) staining. GC was performed using a 2-deoxy glucose based colorimetric GC kit. Protein expression was examined by western blot analysis. PPD, Rg1, Rb2, and Rg3 inhibited lipid accumulation in PA-treated HepG2 cells. PA significantly decreased lipid levels in HepG2 cells, which was prevented by PPD, Rg1, Rb2, and Rg3. PPD, Re, Rb1, and compound K enhanced PA-induced GC inhibition in 3T3-L1 cells, while PPD, Rg3, Rc, and Rh2 enhanced PA-induced GC inhibition in C2C12 cells. PA also significantly decreased the phospho-phosphoinositide 3-kinase, phospho-Akt, phospho- AMP-activated protein kinase α, and phospho-glycogen synthase kinase-3β levels as well as increased glycogen synthase, glucose-6-phosphatase, and phosphoenolpyruvate carboxykinase phosphorylation in all three cell lines, which were prevented by PPD. PPD may be a potential drug candidate that can stimulate GC in key insulin-sensitive tissues, such as the skeletal muscle, liver, and adipose tissue.

## Introduction

Type 2 diabetes (T2D) is a complicated multifactorial disease that has become a dangerous and expensive public health concern worldwide owing to its adverse side effects, including cardiovascular diseases and retinopathy [1]. Insulin resistance (IR) develops in the skeletal muscle, liver, and adipose tissues responsive to insulin before T2D onset. Skeletal muscle, liver, and adipose tissue modulate glucose homeostasis by regulating glucose utilization. IR in tissues is characterized by impaired glycogenolysis, glycogen synthesis, and gluconeogenesis. The liver produces and removes lipids from the body [2]. IR is closely associated with excess dietary lipid accumulation. Ectopic free fatty acid accumulation in hepatocytes impairs insulin signaling. Hepatic lipid overload contributes to hepatic IR progression [3]. Thus, studies on T2D improvement with hepatic lipid overload and hepatic IR development have focused on natural products with the potential to reduce lipid accumulation and regulate glucose utilization [4]. Synthetic anti-diabetic agents such as thiazolidinedione and metformin have been used to improve hepatic IR. However, these drugs are often associated with various adverse reactions such as weight gain, bone loss, and hypoglycemia. Rosiglitazone belongs to the thiazolidinedione class of anti-diabetic agents and inhibits lipid accumulation [5,6]. To date, this anti-T2D drug has not been reported to cause hypoglycemia or hepatotoxic side effects. However, fluid retention is a common side effect of this drug [7]. Therefore, potent anti-T2D drugs with limited side effects should be developed to improve IR.

Various components derived from *Panax ginseng*, such as ginsenosides, improve T2D and IR in diet-induced *in vitro* and *in vivo* obesity models. Nine ginsenosides derived from *P. ginseng*, Rg3, Rb2, Re, Rc, Rh2, Rb1, Rg1, compound K, and protopanaxadiol (PPD), have attracted special attention because of their anti-T2D activity in insulin-responsive tissues. Rg1 exerts anti-T2D effects; improves IR, hepatic function, and blood lipid levels in T2D rats [8,9]; and promotes glucose consumption (GC) by insulin-resistant hepatic [10–12] and muscle cells [13]. Rb2 inhibits hepatic gluconeogenesis in T2D mice and rat hepatocytes [14,15]. Rg3 promotes GC in insulin-resistant muscle cells [16,17] and ameliorates hepatic injury in T2D mice [18]. Re, Rb1, and compound K promote GC in 3T3-L1 pre-adipocytes via insulin receptor substrate-1 (IRS-1) upregulation [19–21]. Rc, Rh2, and Rg3 promote AMPK-mediated GC in C2C12 myotubes [22–24]. However, the effects of ginsenosides on lipid accumulation and palmitic acid (PA)-induced IR in C2C12 myotubes, HepG2 hepatoma cells, and 3T3-L1 adipocytes are not fully understood. Therefore, this study evaluated the effects of nine ginsenosides on PA-induced IR in C2C12 myotubes, HepG2 hepatoma cells, and 3T3-L1 adipocytes.

## Materials and methods

### Materials

The ginsenosides used in this study were derived from the roots of *Panax ginseng* (C. A. Mey.). Ginsenosides Rg3 (Product No. CFN98170), Rb2 (CFN99965), Re (CFN99974), Rc (CFN99973), Rh2 (CFN90340), Rb1 (CFN99964), Rg1 (CFN99967), and compound K (CFN99756) were purchased from ChemFaces (Wuhan, Hubei, China). All eight compounds had ≥ 98% purity as confirmed by high performance liquid chromatography (HPLC) analysis provided by the manufacturer. PPD (PHL89767, purity ≥90%), rosiglitazone (R2408, purity ≥98%), and palmitic acid (P0500, purity ≥99%) were purchased from Sigma-Aldrich (St. Louis, MO, USA). Their purity were confirmed by HPLC analysis provided by the manufacturer. PA was dissolved in distilled water (DW) containing DMSO at 80 °C to prepare a 100 mM stock solution. Bovine serum albumin (Sigma-Aldrich) was dissolved in DW at 50 °C to prepare a 5% (w/v) solution. Both the solutions were mixed and used for assays.

### Cell culture

All cell lines were obtained from the American Type Culture Collection (ATCC, Manassas, VA, USA). C2C12 myotubes (ATCC CRL-1772), HepG2 hepatoma cells (ATCC HB-8065), and 3T3-L1 adipocytes (ATCC CL-173) were maintained in Dulbecco’s modified Eagle’s medium (DMEM; Cellgro, Manassas, VA, USA) supplemented with 10% fetal bovine serum (FBS) and 1% penicillin/streptomycin (Invitrogen Co., Grand Island, NY, USA) in a humidified atmosphere (37 °C, 95% air, 5% CO_2_). The 3T3-L1 cell culture medium was supplemented with 10% fetal calf serum (FCS) instead of 10% FBS.

### Cell viability assay

The cytotoxicity of the nine ginsenosides and rosiglitazone in the three cell lines was determined using an Ez-Cytox cell viability assay kit (Daeil Lab Service Co., Seoul, Korea). The three cell lines were plated in a 96-well plate with or without the nine ginsenosides and rosiglitazone and incubated. After 24 h, cell viability was evaluated by treating the cell lines with Ez-Cytox reagent for 1 h at 37 °C. The optical density of the medium at 450 nm was determined using a microplate reader.

### PA-induced lipid accumulation in HepG2 cells

HepG2 cells were plated in a 96-well plate with or without the nine ginsenosides and rosiglitazone, incubated for 2 h, and then treated with 0.5 mM PA for 24 h. The oil droplets were stained with Oil Red O (ORO) solution. GC and associated protein expression levels were assessed for IR development.

### ORO staining

HepG2 cells were incubated with a fixing solution (paraformaldehyde/saline solution) and stained with ORO solution. After removing the solution, the red pigment was extracted using isopropyl alcohol. The optical density of the eluates at 490 nm was determined using a microplate reader.

### Adipogenic differentiation assays

3T3-L1 pre-adipocytes were plated in six-well plates, maintained in DMEM supplemented with 1% penicillin/streptomycin and 10% FCS until they reached 100% confluence, and then maintained for two more days. At day 0 of adipogenic differentiation, the initial culture medium was changed to DMEM supplemented with 0.5 mM 1-methyl-3-isobutylxanthine, 1% penicillin/streptomycin, 10% FBS, 1 µM dexamethasone, 5 µg/mL insulin, and 1 µM dexamethasone. The existing medium cultured for two days was replaced with DMEM containing 10% FBS, 1% penicillin/streptomycin, 10% FBS, and 5 µg/mL insulin. The medium was replaced with fresh medium every two days. On day 6 of adipogenic differentiation, the culture medium was replaced with DMEM supplemented with 1% penicillin/streptomycin and 10% FBS. PPD was added to the culture medium during adipogenic differentiation from days 0–8.

### Glucose uptake assay

After serum starvation and treatment with or without nine ginsenosides, C2C12, HepG2, and 3T3-L1 cell lines were stimulated with 0.5 mM PA for 24 h. The glucose content of the cell culture supernatant was assessed using an uptake assay kit (Sigma-Aldrich) according to the manufacturer’s recommendations as described previously [25].

### Western blotting analysis

Radioimmunoprecipitation assay buffer (Cell Signaling Technology, Danvers, MA, USA) was used for cell lysis and protein solubilization. Samples (20 μg protein each) were separated by SDS–PAGE and electroblotted on PVDF membranes. The blots were incubated with primary antibodies against IRS-1, p-IRS-1, phosphoinositide 3-kinase (PI3K), p-PI3K, Akt, p-Akt, AMP-activated protein kinase α (AMPKα), p-AMPKα, glycogen synthase kinase-3β (GSK-3β), p-GSK-3β, glycogen synthase, p-glycogen synthase, glucose-6-phosphatase (G6Pase), phosphoenolpyruvate carboxykinase (PCK1/PEPC), glyceraldehyde 3-phosphate dehydrogenase, and secondary antibodies (Cell Signaling Technology) overnight at 4 °C. The blots were treated with ECL Plus Western blotting detection reagent (Amersham Biosciences, Piscataway, NJ, USA). The proteins were detected using a chemiluminescence system (SuperSignal West Pico System; Thermo Scientific).

### Statistical analysis

All experiments were performed in triplicate. All analyses were performed using SPSS Statistics ver. 19.0 (SPSS Inc., Chicago, IL, USA). Non-parametric comparisons were conducted using the Kruskal–Wallis test to analyze the results. Differences were considered statistically significant at *p* < 0.05.

## Results

### Effects of PPD, Rg1, Rb2, and Rg3 on lipid accumulation and GC in HepG2 cells

**Fig 1:**
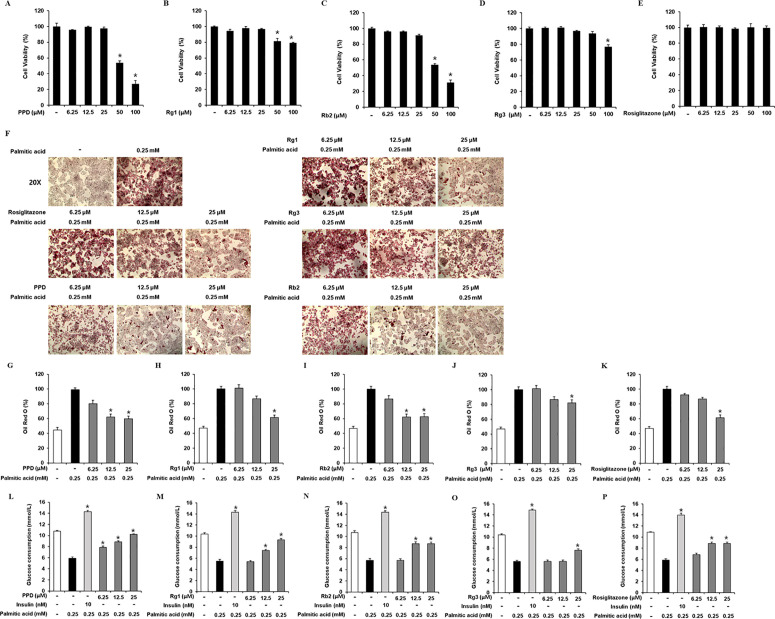
Effects of PPD, Rg1, Rb2, and Rg3 on lipid accumulation and GC in HepG2 cells. Effects of (A) protopanaxadiol (PPD), (B) Rg1, (C) Rb2, (D) Rg3, and (E) rosiglitazone on the viability of HepG2 cells compared to that of the untreated control, as determined via the Ez-Cytox cell viability assay for 24 h. Inhibitory effects of ginsenosides and rosiglitazone on lipid accumulation in HepG2 cells. (F) Images of Oil Red O-stained HepG2 cells treated with palmitic acid (0.25 mM) for 24 h with or without pre-treatment with the indicated concentration of PPD, Rg1, Rb2, Rg3, and rosiglitazone. 4x magnification was used for imaging. (G–K) Level of Oil Red O staining. (L–P) Effects of ginsenosides and rosiglitazone on glucose consumption in HepG2 cells. Glucose consumption in HepG2 cells treated with palmitic acid (0.25 mM) for 24 h with or without pre-treatment with the indicated concentration of PPD, Rg1, Rb2, Rg3, and and rosiglitazone assessed via glucose uptake assay using 2-(N-(7-nitrobenz-2-oxa-1,3-diazol-4-yl) amino)-2-deoxyglucose (2-NBDG). (n = 3 independent experiments, *p < 0.05, Kruskal–Wallis non-parametric test). Data are represented as the mean ± SEM.

The cytotoxicities of PPD, Rg1, Rb2, Rg3, and rosiglitazone (positive control) in HepG2 cells were evaluated (Figs 1A–1E). Up to 25 µM PPD, Rg1, Rb2, Rg3, and rosiglitazone did not affect HepG2 cell viability. Thus, 6.25, 12.5, and 25 μM PPD, Rg1, Rb2, Rg3, and rosiglitazone were chosen for subsequent experiments. In HepG2 cells, PPD (12.5, 25 μM), Rg1 (25 μM), Rb2 (12.5, 25 μM), Rg3 (25 μM), and rosiglitazone (25 μM) inhibited PA-induced lipid accumulation, as measured by ORO staining (Figs 1F–1K). These results suggested that PPD, Rg1, Rb2, and Rg3 decreased PA-induced lipid accumulation in insulin-resistant HepG2 cells. In addition, we determined whether non-toxic concentrations of PPD, Rg1, Rb2, and Rg3 led to an increase in GC. As shown in Figs 1L–1P, 0.25 mM PA significantly decreased the cellular GC than that in the untreated cells, which was prevented by PPD (12.5 and 25 μM), Rg1 (25 μM), Rb2 (12.5 and 25 μM), and Rg3 (25 μM). These results suggest that PPD, Rg1, Rb2, and Rg3 increase GC in PA-induced insulin-resistant HepG2 cells. Considering its influence on lipid accumulation and GC, PPD was selected for the subsequent experiments.

### Inhibitory effects of PPD on gluconeogenic and glycogenic protein expression levels in HepG2 cells

**Fig 2:**
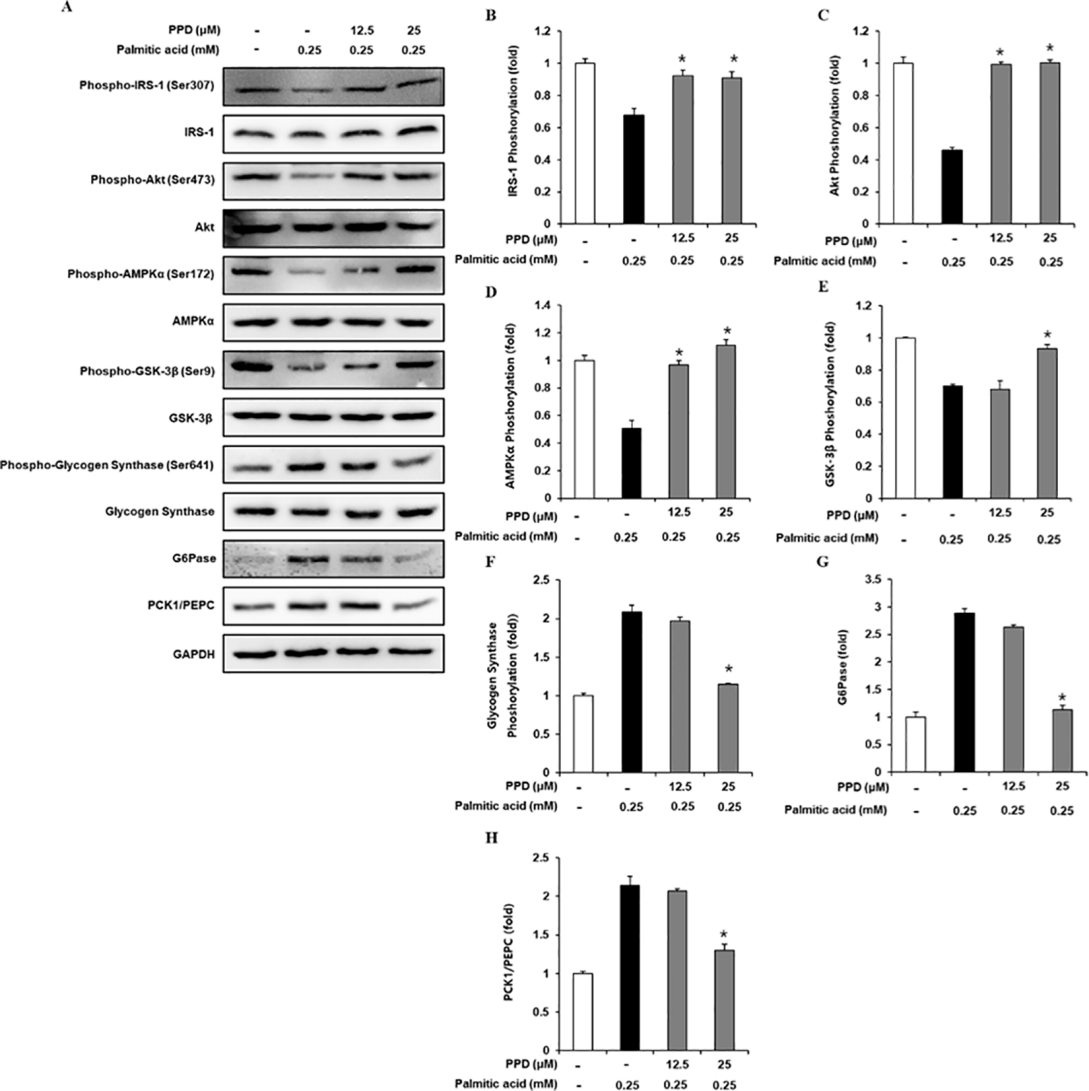
Inhibitory effects of PPD on the expression levels of gluconeogenic and glycogenic proteins in HepG2 cells. (A–H) Protein expression levels and ratios of band intensities of insulin receptor substrate-1 (IRS-1), p-IRS-1, Akt, p-Akt, AMP-activated protein kinase α (AMPKα), p-AMPKα, glycogen synthase kinase-3β (GSK-3β), p-GSK-3β, glycogen synthase, p-glycogen synthase, glucose-6-phosphatase (G6Pase), and phosphoenolpyruvate carboxykinase1/Phosphoenolpyruvate carboxylase (PCK1/PEPC) in HepG2 cells treated with palmitic acid (0.25 mM) for 24 h with or without pre-treatment with the indicated concentration of PPD. (n = 3 independent experiments, *p < 0.05, Kruskal–Wallis non-parametric test). Data are represented as the mean ± SEM.

Western blotting was performed to determine the IRS-1, p-IRS-1, PI3K, p-PI3K, Akt, p-Akt, AMPKα, p-AMPKα, GSK-3β, p-GSK-3β, glycogen synthase, p-glycogen synthase, G6Pase, and PCK1/PEPC expression levels. Treatment with 0.25 mM PA significantly decreased IRS-1, PI3K, Akt, AMPKα, and GSK-3β phosphorylation, which was ameliorated by 12.5 and 25 μM PPD treatment (Fig 2). In addition, glycogen synthase, G6Pase, and PCK1/PEPC phosphorylation significantly increased in 0.25 mM PA-treated cells than that in the untreated cells, which was prevented by 25 μM PPD. These data suggest that PPD increased intracellular glycogen levels by regulating gluconeogenic and glycogenic protein levels in insulin-resistant HepG2 cells.

### Inhibitory effects of PPD, Re, Rb1, and compound K on GC in 3T3-L1 cells

**Fig 3:**
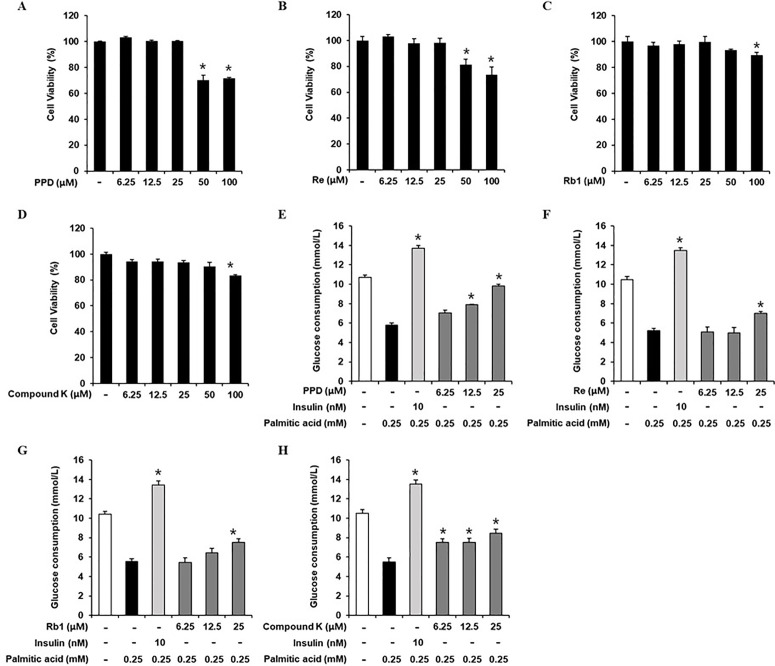
Inhibitory effects of PPD, Re, Rb1, and compound K on glucose consumption in 3T3-L1 cells. Effects of (A) protopanaxadiol (PPD), (B) Re, (C) Rb1, and (D) compound K on the viability of 3T3L1 cells compared to that of the untreated control, as determined via the Ez-Cytox cell viability assay for 24 h. Effects of ginsenosides on glucose consumption in 3T3L1 cells. Glucose consumption in 3T3L1 cells treated with palmitic acid (0.25 mM) for 24 h with or without pre-treatment with the indicated concentration of (E) PPD, (F) Re, (G) Rb1, (H) compound K, and insulin assessed via glucose uptake assay using 2-(N-(7-nitrobenz-2-oxa-1,3-diazol-4-yl) amino)-2-deoxyglucose (2-NBDG). (n = 3 independent experiments, *p < 0.05, Kruskal–Wallis non-parametric test). Data are represented as the mean ± SEM.

The cytotoxicities of PPD, Re, Rb1, and compound K in 3T3-L1 cells were evaluated (Figs 3A–3D). The cell viability assay showed that up to 25 µM PPD, Re, Rb1, and compound K, did not affect 3T3-L1 cell viability. Thus, 6.25, 12.5, and 25 μM PPD, Re, Rb1, and compound K were chosen for subsequent experiments. We determined whether 3T3-L1 cells at non-toxic concentrations led to GC increase. As shown in 3E–H, the cellular GC significantly decreased in 0.25 mM PA-treated cells than that in the untreated cells, which was prevented by 12.5 and 25 μM PPD, 25 μM Re, 25 μM Rb1, and 6.25, 12.5, and 25 μM compound K. These results suggest that PPD, Rg1, Rb2, and Rg3 increase the GC in PA-induced insulin-resistant 3T3-L1 cells. Considering its influence on GC, PPD was selected for subsequent experiments.

### Inhibitory effects of PPD on gluconeogenic and glycogenic protein expression levels in 3T3-L1 cells

**Fig 4:**
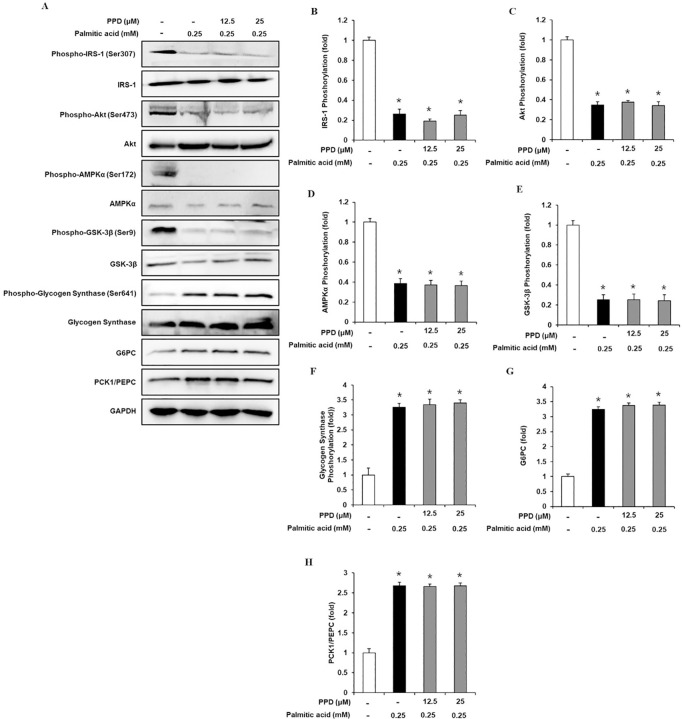
Inhibitory effects of PPD on the expression levels of gluconeogenic and glycogenic proteins in 3T3L1 cells. (A–H) Protein expression levels and ratios of band intensities of insulin receptor substrate-1 (IRS-1), p-IRS-1, Akt, p-Akt, AMP-activated protein kinase α (AMPKα), p-AMPKα, glycogen synthase kinase-3β (GSK-3β), p-GSK-3β, glycogen synthase, p-glycogen synthase, glucose-6-phosphatase (G6Pase), and phosphoenolpyruvate carboxykinase1/Phosphoenolpyruvate carboxylase (PCK1/PEPC) in 3T3L1 cells treated with palmitic acid (0.25 mM) for 24 h with or without pre-treatment with the indicated concentration of PPD (n = 3 independent experiments, *p < 0.05, Kruskal–Wallis non-parametric test). Data are represented as the mean ± SEM.

Western blotting was performed to determine the IRS-1, p-IRS-1, PI3K, p-PI3K, Akt, p-Akt, AMPKα, p-AMPKα, GSK-3β, p-GSK-3β, G6Pase, and PCK1/PEPC expression levels. As shown in Fig 4, IRS-1, PI3K, Akt, AMPKα, and GSK-3β phosphorylation in 0.25 mM PA-treated cells were significantly decreased than that in untreated cells, which was prevented by 12.5 and 25 μM PPD. In addition, 0.25 mM PA treatment significantly increased glycogen synthase, G6Pase, and PCK1/PEPC phosphorylation. However, the expression of all proteins was not altered by PPD treatment.

### Inhibitory effects of PPD, Rg3, Rc, and Rh2 on GC in C2C12 cells

**Fig 5:**
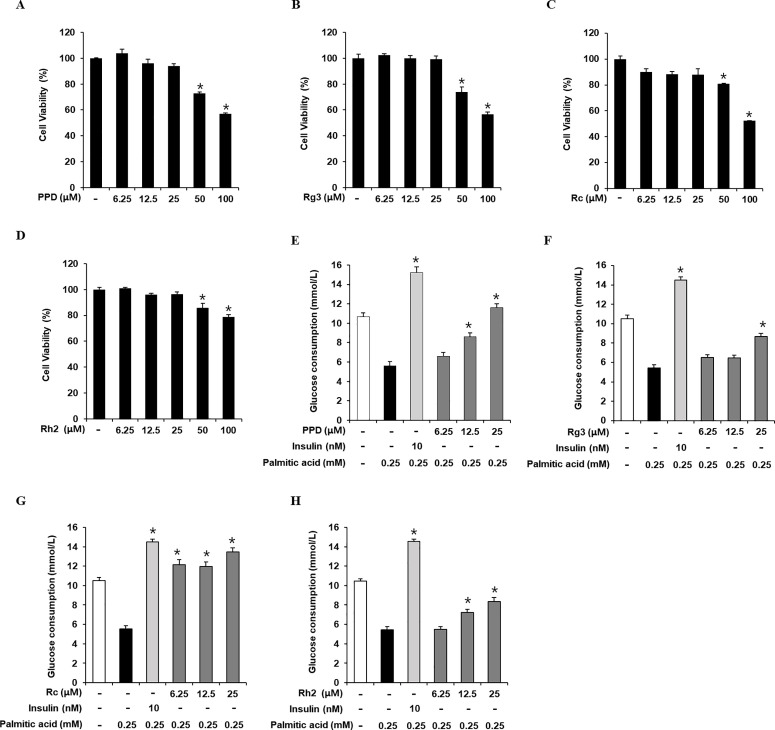
Inhibitory effects of PPD, Re, Rb1, and compound K on glucose consumption in C2C12 cells. Effects of (A) protopanaxadiol (PPD), (B) Rg3, (C) Rc, and (D) Rh2 on the viability of C2C12 cells compared to that of the untreated control, as determined via the Ez-Cytox cell viability assay for 24 h. Inhibitory effects of ginsenosides and rosiglitazone on lipid accumulation in C2C12 cells. Glucose consumption in C2C12 cells treated with palmitic acid (0.25 mM) for 24 h with or without pre-treatment with the indicated concentration of (E) PPD, (F) Rg3, (G) Rc, (H) Rh2, and insulin assessed via glucose uptake assay using 2-(N-(7-Nitrobenz-2-oxa-1,3-diazol-4-yl) amino)-2-Deoxyglucose (2-NBDG) (n = 3 independent experiments, *p < 0.05, Kruskal–Wallis non-parametric test). Data are represented as the mean ± SEM.

The cytotoxicity of PPD, Rg3, Rc, and Rh2 was evaluated in C2C12 cells (Figs 5A-5D). The cell viability assay showed that up to 25 µM, PPD, Rg3, Rc, and Rh2, did not affect C2C12 cell viability. Thus, 6.25, 12.5, and 25 μM PPD, Rg3, Rc, and Rh2 were chosen for subsequent experiments. We determined whether treating C2C12 cells with non-toxic PA concentrations increased. As shown in 5E–H, the cellular GC significantly decreased in 0.25 mM PA-treated cells than that in the untreated cells, which was prevented by 12.5 and 25 μM PPD, 25 μM Rg3, 6.25, 12.5, and 25 μM Rc, and 12.5 and 25 μM Rh2. These results suggest that PPD, Rg3, Rc, and Rh2 increase GC in PA-induced insulin-resistant C2C12 cells. Considering its influence on GC, PPD was selected for subsequent experiments.

### Inhibitory effects of PPD on gluconeogenic and glycogenic proteins expression level in C2C12 cells

**Fig 6:**
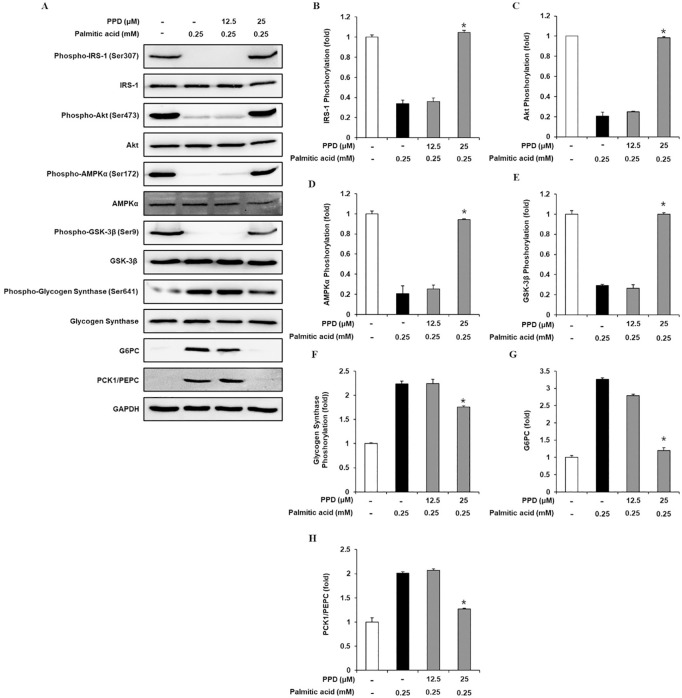
Inhibitory effects of PPD on the expression levels of gluconeogenic and glycogenic proteins in C2C12 cells. (A–H) Protein expression levels and ratios of band intensities of insulin receptor substrate-1 (IRS-1), p-IRS-1, Akt, p-Akt, AMP-activated protein kinase α (AMPKα), p-AMPKα, glycogen synthase kinase-3β (GSK-3β), p-GSK-3β, glycogen synthase, p-glycogen synthase, glucose-6-phosphatase (G6Pase), and phosphoenolpyruvate carboxykinase1/Phosphoenolpyruvate carboxylase (PCK1/PEPC) in C2C12 cells treated with palmitic acid (0.25 mM) for 24 h with or without pre-treatment with the indicated concentration of PPD (n = 3 independent experiments, *p < 0.05, Kruskal–Wallis non-parametric test). Data are represented as the mean ± SEM.

Western blotting was performed to determine the IRS-1, p-IRS-1, PI3K, p-PI3K, Akt, p-Akt, AMPKα, p-AMPKα, GSK-3β, p-GSK-3β, G6Pase, and PCK1/PEPC expression levels. As shown in Fig 6, IRS-1, PI3K, Akt, AMPKα, and GSK-3β phosphorylation were significantly decreased in 0.25 mM PA-treated cells than that in untreated cells, which was prevented by 25 μM PPD. In addition, 0.25 mM PA significantly increased glycogen synthase, G6Pase, and PCK1/PEPC phosphorylation, which was prevented by 25 μM PPD. These data suggested that PPD increased intracellular glycogen levels by regulating gluconeogenic and glycogenic protein levels in insulin-resistant C2C12 cells.

## Discussion

Excessive lipid accumulation within cells causes cell dysfunction, such as lipotoxicity. Saturated fatty acids (stearic and PA) have been reported to induce lipotoxicity [26]. In this study using C2C12 myotubes, HepG2 hepatoma cells, and 3T3 L1 adipocytes, PA was selected to inhibit GC. Treatment with PA in HepG2 cells increases lipid accumulation, which was prevented by PPD, Rg1, Rb2, Rg3, and rosiglitazone. Their effects were similar, which may indicate an imbalance in the lipid metabolism by ginsenosides. The previously reported inhibitory effects of Rg1 [27], Rb2 [28], Rg3 [29], and rosiglitazone on lipid accumulation in in HepG2 cells treated with PA similar to those observed in our study [30]. Rosiglitazone is the member of the thiazolidinedione class known as an oral antidiabetic drug. Rosiglitazone reduces cytotoxicity induced by PA in HepG2 cells and dyslipidemia [30]. In addition, rosiglitazone exerts an inhibitory effect on hepatic fat content and improves the hepatic insulin resistance [31].

Glycogen synthesis and gluconeogenesis are defective in insulin resistance, where glycogen synthesis is inhibited, and gluconeogenesis is enhanced. This manifests as impaired insulin-dependent cellular GC [32]. In the present study, a PA-induced decrease in GC was observed in the three cell lines (C2C12, HepG2, 3T3L1), indicating the possibility of insulin resistance. In HepG2 cells, treatment with PA decreased the GC, while PPD, Rg1, Rb2, and Rg3 improved this effect. Similar to our study, Rg1 improved the PA-induced reduction in GC in HepG2 cells [12]. In HepG2 cells, Rb2 increase the GC [33,34]. In 3T3L1 cells, treatment with PA decreased the GC, while PPD, Re, Rb1, and compound K improved this effect. Similar to our study, Re, Rb1, and Compound K promotes GC in 3T3-L1 preadipocytes via upregulation of insulin receptor substrate-1 (IRS-1) [19–21]. In C2C12 cells, treatment with PA decreased the GC, while PPD, Rg3, Rc, and Rh2 improved this effect. Similar to our study, Rc, Rh2, and Rg3 promote AMPK-mediated GC in C2C12 myotubes [22–24].

Considering GC activity in the three cell lines (C2C12, HepG2, 3T3L1), PPD was selected for subsequent experiments. The results of the western blotting assay confirmed the molecular and cellular mechanisms by which PPD improved PA-induced abnormal gluconeogenesis and glycogen synthesis in the three cell lines (C2C12, HepG2, 3T3L1). In the three key insulin target tissues (skeletal muscle, liver, and white adipose tissue), AMPK phosphorylation is involved in gluconeogenesis and glycogen synthesis, which are impaired in insulin resistance. AMPK activation inhibits gluconeogenesis and enhances GC [35]. Rg1 and Rg3 commonly increase the protein expression of AMPK inhibited by PA in HepG2 cells [27,29]. Rc, Rh2, and Rg3 promote AMPK-mediated GC in C2C12 myotubes [22–24]. Rb1 promote AMPK-mediated GC in 3T3-L1 preadipocytes [36]. Thus, AMPK activation is an attractive target for insulin resistance. Our data showed that, compared to the untreated cells, PA significantly decreased the phosphorylation of AMPKα, which was prevented by PPD in the three cell lines (C2C12, HepG2, 3T3L1). AMPK plays an essential role in GC by up-regulating the IRS-1 pathway.

Upregulation of the IRS-1 pathway in the three key insulin target tissues is related to insulin resistance and impaired glucose metabolism, including gluconeogenesis and glycogen synthesis. In impaired insulin signaling, phosphorylation of IRS-1 is downregulated, followed by a reduction in the downstream PI3K/Akt signaling [37]. PA decreases the phosphorylation of Akt, which can be prevented by Rg1 [12]. Partially consistent with previous results, compared to the untreated cells, PA significantly decreased the phosphorylation of IRS-1/PI3K/Akt in this study, that was prevented by PPD. Our data showed that PPD attenuated PA-induced insulin resistance by regulating the IRS-1/PI3K/Akt signaling pathway in the three cell lines (C2C12, HepG2, 3T3L1). In addition, Akt-mediated phosphorylation of GSK-3β, an important enzyme related to glycogen synthesis, is downregulated in insulin resistance in the three key insulin target tissues. In the basal state of cells, GSK-3β is activated by phosphorylation and inactivates glycogen synthase [38]. The phosphorylation of glycogen synthase, an important enzyme that converts glucose to glycogen, is activated in insulin resistance. In addition, elevated gluconeogenesis is due to insulin resistance, where the gluconeogenic enzymes, PCK1/PEPC and G6Pase, upregulate the IRS-1/PI3K/Akt signaling pathway [39]. In a previous study, PA increased the phosphorylation of G6Pase and PCK1/PEPC, which was prevented by Rg1, respectively [12]. In agreement with previous results, compared to the untreated cells, PA significantly increased the phosphorylation of glycogen synthase, G6Pase, and PCK1/PEPC in this study. These increased proteins were decreased by PPD in the three cell lines (C2C12, HepG2, 3T3L1). All results suggest that PPD may contribute to partial improvement of insulin resistance leading to impaired glucose metabolism, by promoting glycogen synthesis and inhibiting gluconeogenesis in the three cell lines (C2C12, HepG2, 3T3L1).

## Conclusion

The beneficial effects of PPD were evaluated in an in vitro experimental model of PA-induced inhibition of GC. PPD had a better effect on promoting PA-induced inhibition of GC in the three cell lines (C2C12, HepG2, 3T3L1). These beneficial effects were mediated by the modulation of PA-induced abnormal gluconeogenesis and glycogen synthesis by PPD via activation of the AMPK pathway. Our results indicate that PPD may be a potential drug candidate that can stimulate GC in the key insulin-sensitive tissues, skeletal muscle, liver and adipose.

## Supporting information

S1 Raw images. (PDF)

S2 Raw images. (XLSX)
