# Supplementary material for: Protopanaxadiol stimulates glucose consumption by modulating the AMP-activated protein kinase pathway in myotubes, hepatoma cells, and adipocytes
Source: PLoS One. 2025 Jul 29;20(7):e0328486. doi: 10.1371/journal.pone.0328486 (PMC12306732; doi:10.1371/journal.pone.0328486)
Supplement: S1 — (PDF) [file pone.0328486.s001.pdf]

A

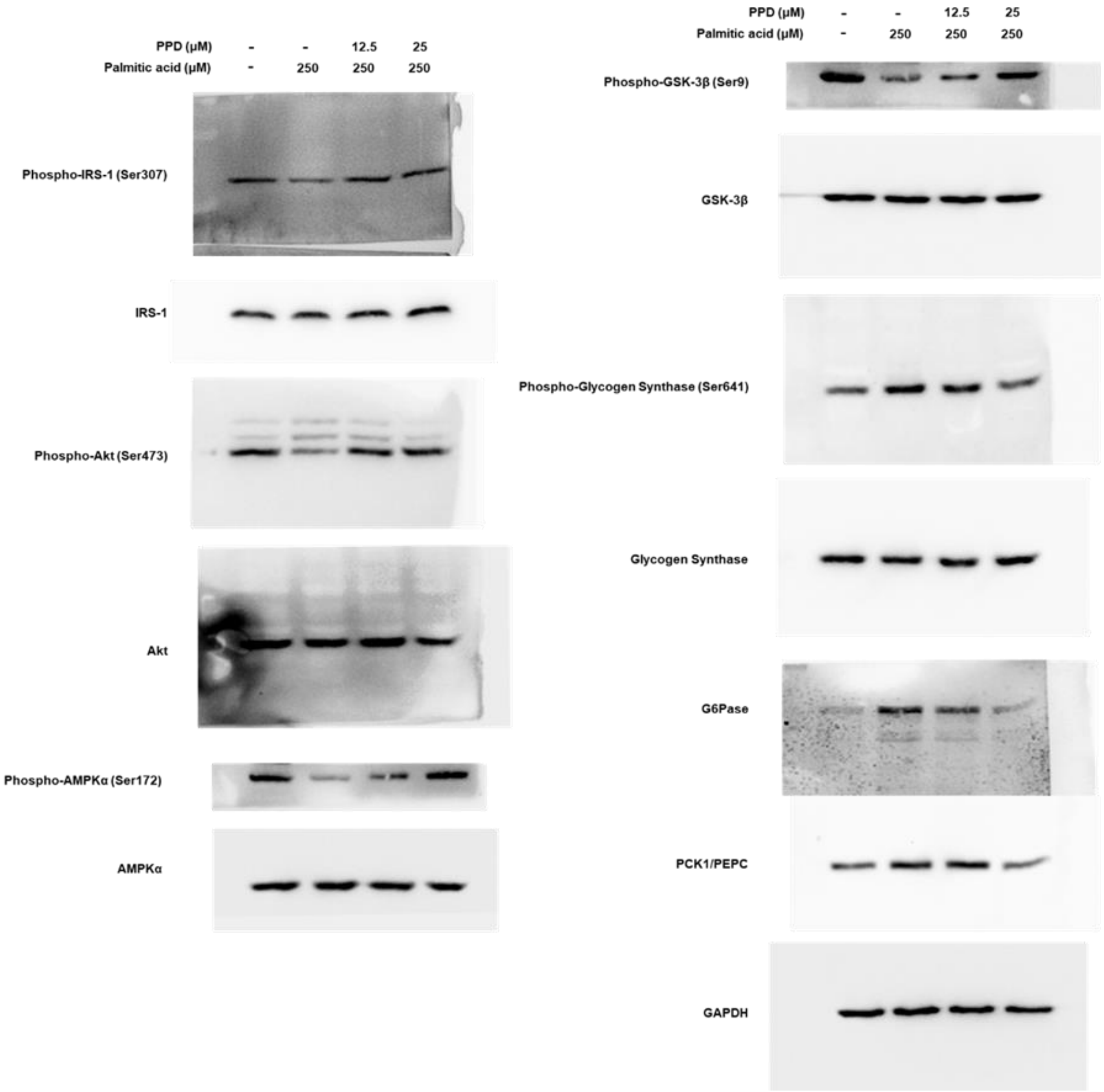

Fig 2. Inhibitory effects of PPD on the expression levels of gluconeogenic and glycogenic proteins in HepG2 cells. (A–H) Protein expression levels and ratios of band intensities of insulin receptor substrate-1 (IRS-1), p-IRS-1, Akt, p-Akt, AMP-activated protein kinase α (AMPKα), p-AMPKα, glycogen synthase kinase-3β (GSK-3β), p-GSK-3β, glycogen synthase, p-glycogen synthase, glucose-6-phosphatase (G6Pase), and phosphoenolpyruvate carboxykinase1/Phosphoenolpyruvate carboxylase (PCK1/PEPC) in HepG2 cells treated with palmitic acid (0.25 mM) for 24 h with or without pre-treatment with the indicated concentration of PPD. (n = 3 independent experiments, *$p < 0.05$, Kruskal–Wallis non-parametric test). Data are represented as the mean ± SEM.

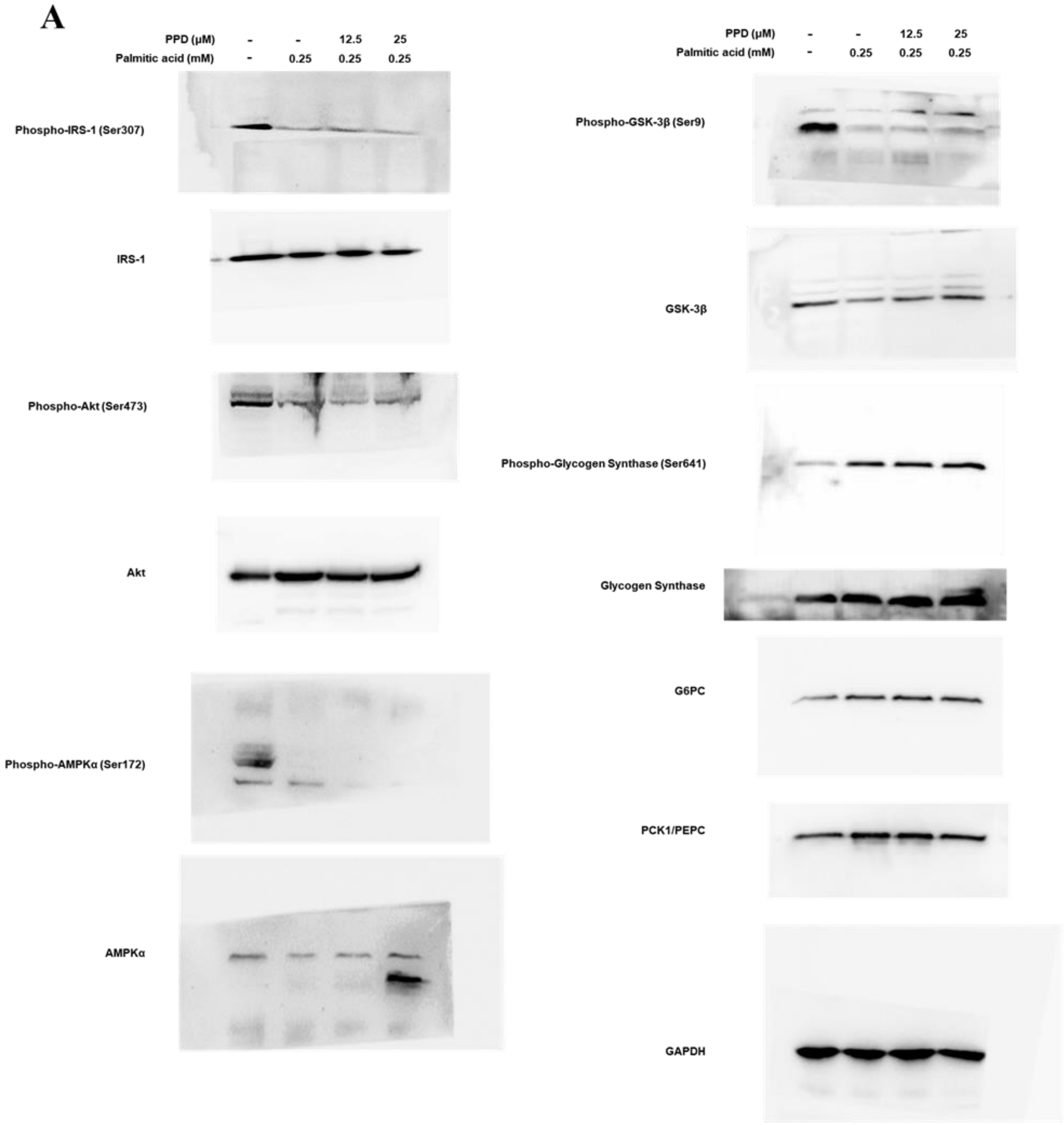

Fig 4. Inhibitory effects of PPD on the expression levels of gluconeogenic and glycogenic proteins in 3T3L1 cells. (A–H) Protein expression levels and ratios of band intensities of insulin receptor substrate-1 (IRS-1), p-IRS-1, Akt, p-Akt, AMP-activated protein kinase α (AMPKα), p-AMPKα, glycogen synthase kinase-3β (GSK-3β), p-GSK-3β, glycogen synthase, p-glycogen synthase, glucose-6-phosphatase (G6Pase), and phosphoenolpyruvate carboxykinase1/Phosphoenolpyruvate carboxylase (PCK1/PEPC) in 3T3L1 cells treated with palmitic acid (0.25 mM) for 24 h with or without pre-treatment with the indicated concentration of PPD (n = 3 independent experiments, *$p < 0.05$, Kruskal–Wallis non-parametric test). Data are represented as the mean ± SEM.

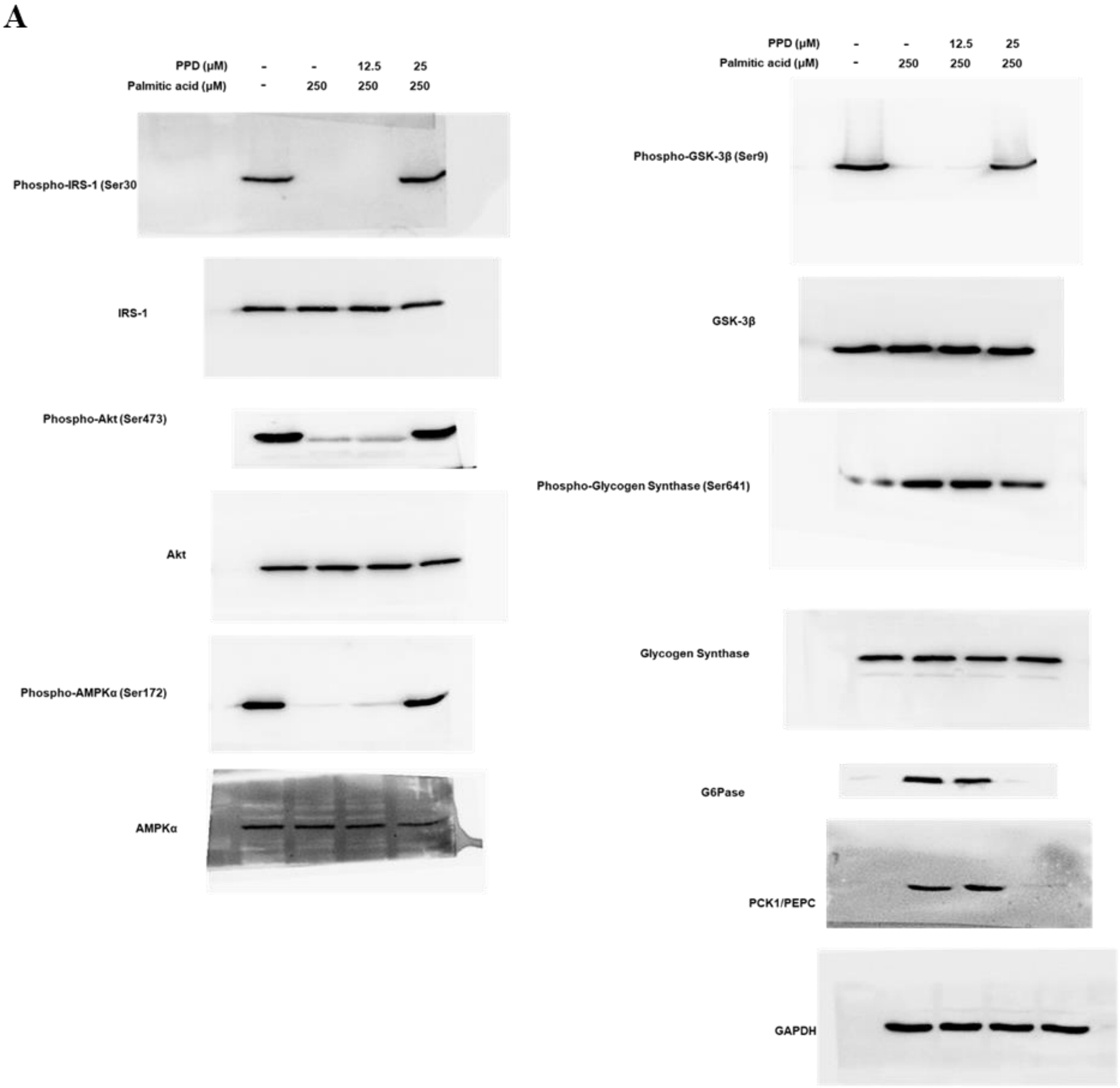

Fig 6. Inhibitory effects of PPD on the expression levels of gluconeogenic and glycogenic proteins in C2C12 cells. (A–H) Protein expression levels and ratios of band intensities of insulin receptor substrate-1 (IRS-1), p-IRS-1, Akt, p-Akt, AMP-activated protein kinase α (AMPKα), p-AMPKα, glycogen synthase kinase-3β (GSK-3β), p-GSK-3β, glycogen synthase, p-glycogen synthase, glucose-6-phosphatase (G6Pase), and phosphoenolpyruvate carboxykinase1/Phosphoenolpyruvate carboxylase (PCK1/PEPC) in C2C12 cells treated with palmitic acid (0.25 mM) for 24 h with or without pre-treatment with the indicated concentration of PPD (n = 3 independent experiments, *$p < 0.05$, Kruskal–Wallis non-parametric test). Data are represented as the mean ± SEM.
